# miRA: adaptable novel miRNA identification in plants using small RNA sequencing data

**DOI:** 10.1186/s12859-015-0798-3

**Published:** 2015-11-05

**Authors:** Maurits Evers, Michael Huttner, Anne Dueck, Gunter Meister, Julia C. Engelmann

**Affiliations:** 1 Institute of Functional Genomics, University of Regensburg, Regensburg, Germany; 2 Biochemistry Center Regensburg (BZR), Laboratory for RNA Biology, University of Regensburg, Regensburg, Germany

**Keywords:** Sequencing data, miRNA identification, RNA secondary structure, *Chlamydomonas reinhardtii*, Small RNA sequencing, Next generation sequencing

## Abstract

**Background:**

MicroRNAs (miRNAs) are short regulatory RNAs derived from longer precursor RNAs. miRNA biogenesis has been studied in animals and plants, recently elucidating more complex aspects, such as non-conserved, species-specific, and heterogeneous miRNA precursor populations. Small RNA sequencing data can help in computationally identifying genomic loci of miRNA precursors. The challenge is to predict a valid miRNA precursor from inhomogeneous read coverage from a complex RNA library: while the mature miRNA typically produces many sequence reads, the remaining part of the precursor is covered very sparsely. As recent results suggest, alternative miRNA biogenesis pathways may lead to a more diverse miRNA precursor population than previously assumed. In plants, the latter manifests itself in e.g. complex secondary structures and expression from multiple loci within precursors. Current miRNA identification algorithms often depend on already existing gene annotation, and/or make use of specific miRNA precursor features such as precursor lengths, secondary structures etc. Consequently and in view of the emerging new understanding of a more complex miRNA biogenesis in plants, current tools may fail to characterise organism-specific and heterogeneous miRNA populations.

**Results:**

miRA is a new tool to identify miRNA precursors in plants, allowing for heterogeneous and complex precursor populations. miRA requires small RNA sequencing data and a corresponding reference genome, and evaluates precursor secondary structures and precursor processing accuracy; key parameters can be adapted based on the specific organism under investigation. We show that miRA outperforms the currently best plant miRNA prediction tools both in sensitivity and specificity, for data involving *Arabidopsis thaliana* and the Volvocine algae *Chlamydomonas reinhardtii*; the latter organism has been shown to exhibit a heterogeneous and complex precursor population with little cross-species miRNA sequence conservation, and therefore constitutes an ideal model organism. Furthermore we identify novel miRNAs in the Chlamydomonas-related organism *Volvox carteri*.

**Conclusions:**

We propose miRA, a new plant miRNA identification tool that is well adapted to complex precursor populations. miRA is particularly suited for organisms with no existing miRNA annotation, or without a known related organism with well characterized miRNAs. Moreover, miRA has proven its ability to identify species-specific miRNAs. miRA is flexible in its parameter settings, and produces user-friendly output files in various formats (pdf, csv, genome-browser-suitable annotation files, etc.). It is freely available at https://github.com/mhuttner/miRA.

**Electronic supplementary material:**

The online version of this article (doi:10.1186/s12859-015-0798-3) contains supplementary material, which is available to authorized users.

## Background

MicroRNAs (miRNAs) are short endogenous non-coding RNA molecules that play an important role in regulating gene expression in many species within the animal and plant kingdoms. Since the discovery of miRNAs in *Caenorhabditis elegans* [1, 2], detailed studies into transcription and the functional role of miRNAs across different species have lead to a complex picture of miRNA biogenesis and miRNA-associated regulatory pathways [3–5].

### A brief overview of miRNA biogenesis in plants

The first step in miRNA biogenesis involves transcription of a primary miRNA transcript by RNA polymerase II. In canonical miRNA biogenesis, primary transcripts are then processed by the Dicer-like protein DCL1 to produce miRNA precursors (pre-miRNA) [6, 7]. Precursors exhibit double-stranded hairpin structures of varying length and levels of complexity (bulges, multiple shorter sub-hairpins etc.). While animal miRNA precursors are usually 80−100 nt long and consist of simpler hairpin structures, plants and algae have a more heterogeneous precursor population, with pre-miRNAs of up to a few hundred nucleotides in length and often including additional shorter hairpins [6, 8]. Following precursor formation, the pre-miRNA is exported to the cytoplasm, and processed by Dicer-like proteins to a 20−24 nt long double-stranded RNA complex. Either the 5’ or the 3’ arm of the duplex may then be incorporated into the RNA-induced silencing complex (RISC), where it binds to a member of the AGO protein family [9, 10].

### Computational miRNA identification

Computational miRNA identification based on next-generation sequencing (NGS) data involves identifying the genomic location of miRNA precursors, using small RNA expression primarily from the mature miRNA. Small RNA sequencing libraries typically also contain expression from other non-miRNA RNA species, such as other small RNAs with similar lengths and/or degradation products from protein-coding genes; computational miRNA identification requires filtering of these “background” signals in the sequencing data from sequencing reads associated with true miRNA expression. A common approach to do so is to use the fact that many miRNAs are evolutionarily conserved from species to species within the plant and the animal kingdoms. This gives rise to cross-species homologous miRNA families [11]. Database-supported computational tools for identifying novel miRNAs from sequencing data commonly apply a combination of (i) evaluating miRNA secondary structures, and (ii) ranking miRNA candidates by utilising existing annotation or evolutionary conservation of the mature miRNA sequence. Structure threshold parameters of most algorithms are often optimised based on animal miRNA precursor structures. Recent quantitative comparisons of the performance of various existing miRNA identification algorithms are given in [12] and [13].

Recent studies suggest that miRNA precursors often show more complex features and secondary structures, such as multiple mature/star duplexes per precursor, multiple hairpin loops, and tRNA precursor-like clover structures [14, 15]. In plants, reports of mature miRNAs of different lengths (21 nt, 22 nt and 24 nt) originating from longer (up to a few hundred nt) long precursors have shown that species-specific (non-conserved) miRNAs exist (see e.g. [16] and [17]), and play an important role in developing a better understanding of mechanisms related to miRNA origin and evolution [6, 18, 19].

Here we introduce miRA for identifying miRNA precursors in plants and plant-like organisms (algae). miRA requires aligned small RNA sequencing data and a reference genome, and does not depend on existing miRNA annotation. Its main strength lies in the identification and charactersation of complex and non-homogeneous miRNA precursor populations. To our knowledge, miRA is also the first tool that allows to identify expression from multiple mature miRNA loci within one precursor. Within miRA, miRNA precursors are identified based on a set of species-specific constraints. Two key aspects of the algorithm presented in this paper are (1) not requiring cross-species miRNA sequence conservation, and (2) allowing for a heterogeneous miRNA precursor population. This allows for a consistent characterisation of species-specific miRNAs and heterogeneous miRNA precursor structures in plants and algae, which in turn provides insight into the role of non-canonical miRNA biogenesis in these organisms.

We compare the performance of miRA with popular miRNA prediction tools using NGS data from two different organisms (*Arabidopsis thaliana*, *Chlamydomonas reinhardtii*), and identify novel miRNAs in the *Chlamydomonas reinhardtii*-related Volvocine algae *Volvox carteri*. The latter two organisms show a high degree of genome similarity, with recent results suggesting (1) very little conservation between miRNAs identified in both organisms, and (2) the existence of a heterogeneous miRNA precursor population [20, 21]. Both organisms therefore constitute an ideal example to apply and evaluate our algorithm. Results show an absence of miRNA conservation between both organisms, suggesting profoundly different, evolutionary-specific roles of miRNAs in *Chlamydomonas reinhardtii* and *Volvox carteri*.

## Implementation

miRA uses high-throughput RNA sequencing data (typically small RNA sequencing data), and relies on a genome-wide investigation of secondary hairpin structures. For reasons detailed in the introduction, we choose to be independent of cross-species sequence conservation. Therefore the process of identifying novel miRNAs depends (1) on the identification of a secondary structure that is consistent with that of a miRNA precursor, and (2) on a miRNA candidate precursor verification based on a precursor processing and read-coverage analysis.

Core modules of miRA are written in the C programming language, making full use of thread parallelisation on multi-core architectures using OpenMP [22]. miRA compiles and runs on any UNIX-based architecture (Linux, Mac OS X). To use miRA optimally, java, gnuplot and LATE X should be installed. miRA is implemented using test-driven development, allowing every program function to be tested for its correct behaviour upon compilation of the source code, using the open-source MIT-licensed unit testing library ’testerino’. miRA includes customised versions of the RNAfold [23] and Varna [24] libraries, which were modified to allow the extraction of relevant data and remove unused code.^1^

Output files and plots are automatically generated, including (i) a GTF- and BED-formatted (see e.g. [25] for a description of the file formats) list of identified miRNA precursors and mature/star miRNAs for use in common genome browsers, (ii) a LATE X-based PDF report including secondary structure plots for every identified miRNA precursor, and (iii) a HTML-formatted table of all identified miRNA precursors including links to full miRNA reports that can be viewed in a web browser. The code can be downloaded from github https://github.com/mhuttner/miRA. Documentation and example files are included. The user typically runs miRA by specifying the species-group under consideration (i.e. plants, algae). Alternatively the user may adjust key parameters individually. The modular structure of miRA enables the user to restart different sections of the pipeline. This allows for efficient computer time and resource management, in particular for jobs involving large genomes and/or sequencing data.

## Method

In a three-stage process, we first identify genomic contigs based on small RNA sequencing data. In the second step, we analyse secondary structures for every cluster. Lastly, we verify that RNA sequencing data-based read coverage of miRNA precursor candidates is consistent with miRNA precursor processing resulting in the expression of one or more mature/star miRNA duplexes. We give details involving each step and a discussion of important key parameters (typeset in sans-serif) in the following sections^2^. Note that the time-consuming step of folding candidate sequences can be parallelised on multiple computer core architectures, by adjusting the parameter openmp_thread_count. If the OpenMP library is not present, this parameter will be ignored, and a single thread will be used for the sequence folding.

### Defining candidate clusters

**Fig. 1:**
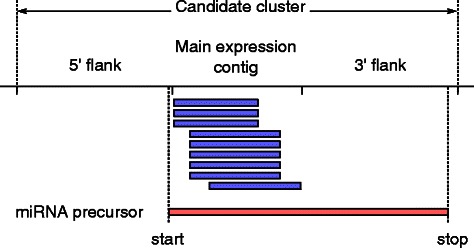
Diagram illustrating the definition of a candidate cluster based on the main expression contig extended by a flanking region on the 5^′^ and 3^′^ end. Reads of the main expression contig are shown by the blue bars. The potential miRNA precursor with start/stop coordinates in the 5’/3’ flanking region is shown in red

We require aligned strand-specific (small) RNA sequencing data in form of a sequence alignment/map (SAM) file, and a FASTA-formatted reference genome. In a first step towards identifying novel miRNA precursors, we generate a list of genomic regions based on and centred around a confined locus (contig) exceeding a threshold number of aligned and overlapping reads (cluster_min_reads). The latter was fixed at 10 reads for the analysis presented in this paper. This main expression contig is then extended at the 5^′^ and 3^′^ ends by an *F*=200 nt (default for cluster_flank_size) long flanking region, thus forming the candidate cluster as shown in Fig. 1. The length of the 5’/3’ end flank should be chosen such that candidate clusters are at least as long as miRNA precursors in the organism under investigation. The default value for cluster_flank_size should be suitable for most plant and plant-like organisms. Prior to extending contigs by the flanking regions, we merge neighbouring expression loci that lie less than 10 nucleotides (default for cluster_gap_size) apart, to form one combined contig. Finally we discard contigs exceeding a length of 2000 nt (default for cluster_max_length).

### Secondary structure investigation

In the second step of the analysis, we investigate secondary structures that result from folding sequences of different lengths (i.e. different start and stop positions) located within the candidate cluster locus as defined in the previous analysis step, see Fig. 1. We require miRNA precursors to fulfil the following key criteria:

1. Existence and uniqueness of one optimal (i.e. minimal in its free energy) structure amongst all possible structures (c.f. [26]), the corresponding sequences of which have genomic start/stop coordinates located within the cluster’s 5^′^/ 3^′^ flanking region.
2. A set of species-dependent secondary structure constraints, detailed in the following sections.
3. Statistical significance of the obtained optimal structure compared to structures resulting from random sequences with the same length and nucleotide distribution.

#### Minimum in the secondary structure free energy surface

Candidate cluster regions are folded using a modified version of RNALfold [23]. We calculate per-nucleotide (i.e. sequence length-normalised) minimum free energies MFE/nt for sequences with different start/stop coordinates within the 5^′^/ 3^′^ flanking region. The optimum sequence corresponds to the structure with the lowest MFE/nt.

#### Secondary structure constraints

We filter sequences corresponding to optimal secondary structures based on structure constraints that are consistent with characteristic features of miRNA precursors in the organism under investigation. Relevant key parameters are

1. the per-nucleotide minimum free energy of the secondary structure ’ MFE/nt’ (min_mfe_per_nt),
2. the number of terminal loops ’ *N*_term_’ (max_hairpin_count), and
3. the length in nucleotides of the longest double-stranded segment allowing for two mismatches ’ *L*_ds,max_’ (min_double_strand_length) within the candidate structure.

It is important to note that these parameters are not necessarily independent of each other, as e.g. an increase in *L*_ds,max_ leads to a smaller minimum free energy MFE/nt of the resulting secondary structure.

**Fig. 2:**
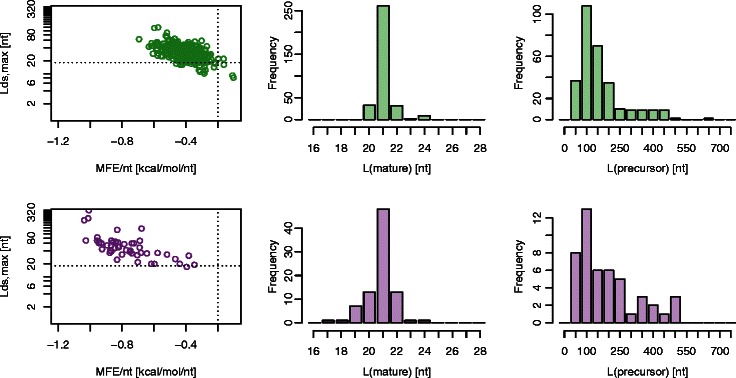
Distributions of the minimum free energy per nucleotide of the miRNA precursor secondary structure (MFE/nt), length of longest double-stranded segment within the precursor (*L*_ds,max_) and length of the precursor (*L*(precursor)) for known miRNA precursors in *Arabidopsis thaliana* (*top* three panels) and *Chlamydomonas reinhardtii* (*bottom* three panels). All precursor sequences were obtained from miRBase ([27]). Secondary structure calculations were performed using RNAfold ([23]). The dotted lines mark the thresholds adopted for the identification of miRNAs in the corresponding organisms

We determined key parameters for different organisms based on an analysis of secondary structures of known miRNA precursors. To this extent, microRNA precursor sequences were obtained from miRBase [27], and their corresponding optimal secondary structures were analysed. We show the distribution of the minimum free energy per nucleotide of the miRNA precursor secondary structure (MFE/nt), length of the longest double-stranded segment within the precursor (*L*_ds,max_), and length of the precursor (*L*(precursor)) for *Arabidopsis thaliana* (Arabidopsis) and *Chlamydomonas reinhardtii* (Chlamydomonas) in Fig. 2. In comparison to miRNAs in Arabidopsis, Chlamydomonas miRNA precursors have on average longer double-stranded segments, smaller per nucleotide minimum free energies, and a precursor population with a larger variation in lengths.

The mean per-nucleotide minimum free energy decreases inversely with the sequence length *L* as $\text {MFE/nt} \propto 1/L$. This initially rapid decrease of MFE/nt with increasing sequence length may lead to precursor structures that include additional short maximally paired hairpins, in particular in organisms with longer precursors (plants, algae). While these sub-hairpins are not necessarily biologically realistic, the main structure without these extra hairpin(s) may still be consistent with that of a miRNA precursor. By choosing the number of terminal loop hairpins in the potential precursor structure to be *N*_term_<4, we assure that such structures are not discarded prematurely.

#### Statistical significance test

In a next step, the statistical significance of sequences which pass all structure constraints is investigated. To obtain a statistical measure (*p*-value) related to the significance of the secondary structure, we determine null distributions of the per-nucleotide minimum free energy *f*(MFE/nt) for every sequence that passes the structure constraints filter (max_pvalue). This is done by randomly permuting nucleotides of the sequence using the Fisher-Yates algorithm (mono-nucleotide shuffling), and calculating corresponding minimum free energies. We account for sequence-specific nucleotide abundances by calculating null distributions for every sequence separately. The *p*-value related to the significance of the per-nucleotide minimum free energy MFE/nt of the candidate sequence is then obtained from

$$p = \int_{-\infty}^{\text{MFE/nt}}d(\text{MFE/nt}')f(\text{MFE/nt}')\,. $$

For the calculation of the structure significance *p*-value no assumption is made upon the nature of the distribution; however it is interesting to note that the distribution of MFE/nt for random sequences is fairly well approximated by a normal distribution. Di-nucleotide shuffling does not lead to different results. This can be attributed to the fact that mono-nucleotide and di-nucleotide shuffling of longer sequences (${\gtrsim 100\,\text {nt}}$) lead to the same distribution of MFE/nt. Results of the structure significance analysis for two candidate sequences are summarised in Figure S1 of the Additional file 1.

### miRNA precursor verification

In the last step of the analysis pipeline, we use a read coverage-based verification procedure to investigate whether observed expression from the miRNA precursor locus is consistent with that of a miRNA precursor containing at least one mature miRNA. The verification process consists of a series of adjustable constraints on the identified mature/star miRNA sequences, which are related to precursor processing accuracy. For each miRNA precursor candidate, we validate that

1. a mature miRNA locus can be defined with the following properties:
  - Sharp edges in strand-specific read coverage at the 5^′^ or 3^′^ end, containing >min_coverage of the full miRNA precursor coverage.
  - Length min_duplex_length≤*L*(mature)<max_duplex_length, and
2. taking into account DCL processing leading to 2 nt 3’ overhangs, a complementary star miRNA segment of length min_duplex_length≤*L*(star)<max_duplex_length exists.

Additionally, we may require that (adopted from [8]):

3. the fraction of paired nucleotides within the mature miRNA locus is ≥min_paired_fraction,
4. the mature miRNA segment does not fold back on itself, and
5. the mature miRNA segment has <4 adjacent unpaired nucleotides at nucleotide positions 3…*L*(mature)−3 (allow_three_mismatches), and <3 adjacent unpaired nucleotides at the 5^′^ and 3^′^ end of the duplex (allow_two_terminal_mismatches).

We give a list of adopted key parameters for different organisms in Table S1 of the Additional file 1. Parameters are consistent with values discussed in various other publications such as [8, 28].

The underlying alignment data used in the verification process can be different from the alignment data used for the initial identification of main expression contigs. This allows for an independent cross-verification of candidate miRNAs.

## Results and discussion

We perform a benchmark analysis of miRA and up-to-date, plant-suitable sequence data-based miRNA prediction tools. We use the prediction tools miRDP (formerly called miRDeep-P) [29] and miR-PREFeR [28], the latter of which was demonstrated to show superior performance compared to other existing tools [28]. Attempts to use miRExpress [30] and miReNA [31] were unsuccessful: miRExpress failed to compile on recent Linux and Mac OS X builds, and miReNA failed to identify any miRNA from the NGS data; while these tools have demonstrated their applicability in regards to miRNA identification, results highlight the need for a flexible and easy-to-use miRNA identification method such as miRA.

We use both simulated and experimental data to evaluate and compare the performance of miRA and miR-PREFeR. For each method and data set we determine the recall rate (i.e. sensitivity or true positive rate) RR=TP/(TP+FN), where TP and FN are the number of true positives and false negatives, respectively. Additionally, the analysis of results based on the simulated data allows us to investigate the specificity (i.e. true negative rate) SPC=TN/(FP+TN), where TN and FP are the number of true negatives and false positives, respectively. Details and results involving the different data sets are given in the following sections.

### Simulated data

We simulate miRNA and background expression from protein-coding genes (the latter constituting false positives) of the Volvocine algae *Chlamydomonas reinhardtii* (Chlamydomonas) using Flux Simulator [33]. For 20 known miRNA precursors with unambiguous strand-assignment from [8], we generate reads for the mature and star loci. Expression strengths of mature/star loci within the precursors, as well as expression strengths of the precursors themselves are sampled uniformly. For the latter we choose a minimum expression strength to make sure that all miRNAs are expressed. The set of Flux Simulator parameters is given in Table S2 of the Additional file 1. Expression of protein-coding genes is generated using the catalogue of 14,595 annotated protein-coding genes for the Chlamydomonas reference genome version 3.0 from [34]. Simulated reads were then mapped to the Chlamydomonas reference genome [34] using tophat/bowtie2 [32, 35]. The reference genome version matches the version used for annotating miRNAs in [8]. It is important to emphasize that the simulated data set corresponds to a challenging scenario, constituting a high background of degraded transcripts from protein-coding genes and only a small number of miRNAs.

It is interesting to note that annotated Chlamydomonas miRNAs constitute a heterogeneous precursor population (see [8]), with 50 of all precursors having more than two 21 nt long main expression loci. Furthermore, folding of most annotated precursor sequences yields minimum energy-associated secondary structures with complex features such as e.g. additional shorter hairpin loops, additional bulges etc. Therefore simulated Chlamydomonas data provides an excellent test data set to investigate the performance of miRNA identification algorithms given a complex miRNA population.

We determine recall rates and specificities of miRA, and compare results with those of miR-PREFeR. Results are summarised in Table 1. miR-PREFeR fails to identify any of the miRNAs.

**Table 1:** Comparison of recall rates (RR) of different NGS-based miRNA identification tools using various data sets

Organism and	Identification	miRNA reference data		*N*_recall_	RR	*N*_tot_	SPC
library reference	method	Source	*N*_ref_				
*Chlamydomonas reinhardtii*							
Simulated	miRA	Molnar et al. [8]	20	12	0.60	19	1.0
Simulated	miR-PREFeR	Molnar et al. [8]	20	0	0.00	0	1.0
Loizeau et al. [36]	miRA	Molnar et al. [8]	47	39	0.83	281	–
Loizeau et al. [36]	miRDP	Molnar et al. [8]	47	14	0.30	964	–
Loizeau et al. [36]	miR-PREFeR	Molnar et al. [8]	47	29	0.62	60	–
Molnar et al. [8]	miRA	Molnar et al. [8]	15	12	0.80	175	–
Molnar et al. [8]	miRDP	Molnar et al. [8]	15	7	0.47	51	–
Molnar et al. [8]	miR-PREFeR	Molnar et al. [8]	15	3	0.20	6	–
*Arabidopsis thaliana*							
Pooled Athl-2 [28]	miRA	miRBase	246	122	0.50	517	–
Pooled Athl-2 [28]	miRDP	miRBase	246	80	0.12	695	–
Pooled Athl-2 [28]	miR-PREFeR	miRBase	246	119	0.48	138	–
*Volvox carteri*							
Novel data	miRA	–	0	–	–	213	–

We compare the performance of miRA, miRDP [29], and miR-PREFeR [28] using simulated and experimental algae NGS data (*Chlamydomonas reinhardtii* and *Volvox carteri*), and *Arabidopsis thaliana* NGS data. Details of the simulated data are given in the text. We determine the number of reference miRNAs for each library by requiring a minimum expression of 10 reads for each known reference miRNA. The source and number *N*_ref_ of known miRNAs for the different organisms are given in columns 3 and 4. *N*_recall_ gives the number of identified known miRNAs. *N*_tot_ gives the total number of identified miRNAs. For the simulated data we provide the specificity (SPC) in the last column

### Experimental data

#### Chlamydomonas reinhardtii

We use two different Chlamydomonas small RNA sequencing libraries from [36] and [8]. Corresponding adapter-trimmed and quality-filtered sequencing data were obtained from the gene expression omnibus (GEO), accession numbers GSE32457 (http://www.ncbi.nlm.nih.gov/geo/query/acc.cgi?acc=GSE32457) and GSE7575 (http://www.ncbi.nlm.nih.gov/geo/query/acc.cgi?acc=GSE7575), and converted to the FASTA format. Resulting reads were again mapped to the Chlamydomonas reference genome version 3.0 using tophat/bowtie2. The reference genome version was chosen such that results allowed for a direct comparison of derived miRNA loci with those from [8].

We identify Chlamydomonas miRNA precursors using miRA, and determine recall rates based on the list of known miRNAs from [8]. We use Molnar’s list of annotated miRNAs instead of Chlamydomonas data from miRBase due to the existence of duplicate entries in the miRBase data, primarily from [20]. Molnar et al. list 31 Chlamydomonas miRNAs with unambiguous precursor strands, and 19 miRNAs with ambiguous precursor strands. We exclude miRNA precursors from the lists that are located on unassembled bonus scaffolds since we do not include these extra scaffolds in our Chlamydomonas reference genome. The final number of reference miRNAs used for calculating recall rates is determined by requiring a minimum expression of 10 reads per known reference miRNA, and the resulting numbers are given in Table 1.

**Fig. 3:**
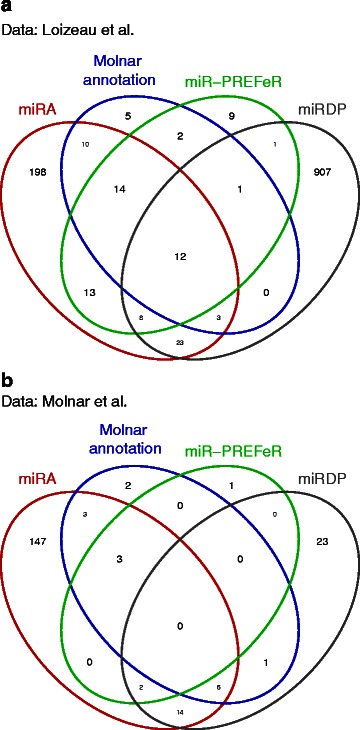
Venn diagrams showing the intersection of predicted miRNAs based on different tools for the two different *Chlamydomonas reinhardtii* sequencing datasets: Panel (**a**) [36], Panel (**b**) [8]. The reference annotation is based on the list of annotated miRNAs from Molnar et al. [8]. Details involving the experimental and reference data are given in the text and in Table 1

Results based on miRA, miRDP and miR-PREFeR for both data sets are summarised in Table 1. miRA recall rates for both data sets are comparable and ≥80 *%*. The larger number of novel miRNAs derived from the data in [36] compared to those from [8] is related to the larger sequencing depth of the former. This difference in sequencing depth is also reflected in the different numbers of expressed reference miRNAs. Recall rates for mRDP and miR-PREFeR are significantly smaller, dropping well below 50 % in some cases; in a direct comparison of miRDP and miR-PREFeR, the former seems to perform better with low sequencing depth data, while miR-PREFeR outperforms miRDP with deeper sequencing data. A detailed comparison of identified miRNAs using miRA, miRDP and miR-PREFeR is given in Fig. 3.

We are able to identify many novel Chlamydomonas miRNA precursors, many of which show expression profiles consistent with the generation of two and more mature miRNAs from the same precursor. Corresponding precursor structures range from up to 500 nt long single hairpin structures, and up to 700 nt long multiple hairpin structures. Examples of complex miRNA precursors are given in Figure S2 of the Additional file 1. Structural features of novel miRNA precursor structures often include multiple bulges and larger terminal loops; these complex structures in connection with multiple mature miRNA expression and overall longer precursors are believed to be responsible for miR-PREFeR and miRDP not being able to successfully identify the corresponding expressed loci with potential miRNA precursors.

**Fig. 4:**
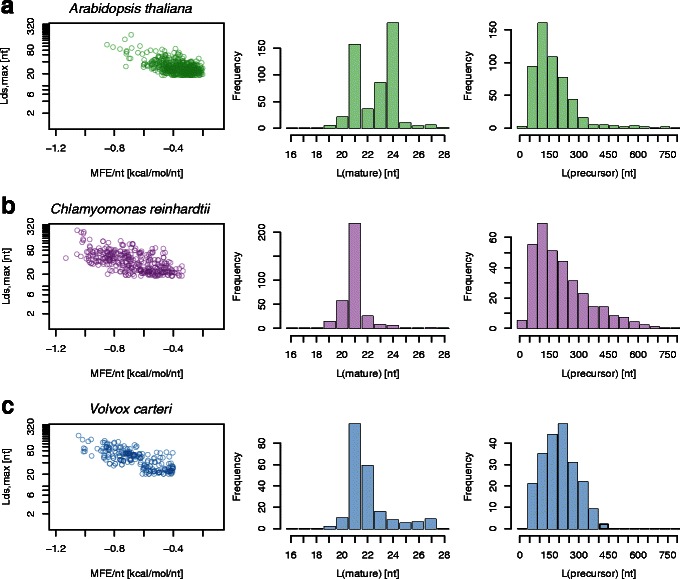
Distributions of per-nucleotide minimum free energy (MFE/nt), length of the longest double-stranded segment within the miRNA precursor (*L*_ds,max_), length of the primary (i.e. most strongly expressed) mature miRNA (*L*(mature)), and length of the miRNA precursor *L*(precursor) for the verified miRNA precursors in *Chlamydomonas reinhardtii* (*top panel*) and *Volvox carteri* (*bottom panel*) following analysis of small RNA sequencing data

A complete list of identified Chlamydomonas miRNA precursors is provided in Table S3 of the Additional file 1. Distributions of the key parameters per-nucleotide minimum free energy of the miRNA precursor secondary structure (MFE/nt), length of the longest double-stranded segment in the precursor (*L*_ds,max_), length of the primary (i.e. most strongly expressed) mature miRNA (*L*(mature)), and length of the miRNA precursor (*L*(precursor)) for the identified (known and novel) Chlamydomonas miRNA precursors based on the data from [36] are summarised in the three middle panels of Fig. 4. They show good agreement with corresponding distributions derived from miRBase Chlamydomonas miRNA precursors as shown in the bottom panel of Fig. 2. We see from Fig. 4 that precursor lengths vary significantly, extending to up to ∼700 nt. Corresponding secondary structures confirm the existence of a complex and heterogeneous miRNA precursor population.

### Arabidopsis thaliana

We use an *Arabidopsis thaliana* (Arabidopsis) library containing two samples that was used in the miR-PREFeR publication [28]. Details of the Athl-2 datasets can be found in the supplements of [28]. Pooled reads were mapped to the TAIR10 reference genome [37] using tophat/bowtie2. A list of known reference miRNAs was downloaded from miRBase and filtered by requiring a minimum expression of 10 reads per miRNA.

We compare the performance of miRA, miRDP and miR-PREFeR in Table 1. Recall rates of miRA and miR-PREFeR are near identical, with miRA predicting more novel miRNAs. This is believed to be related to miR-PREFeR’s requirement of the existence of star-sequence associated reads, whereas miRA does not impose a minimum expression threshold on the star sequence. We show the distribution of key parameters MFE/nt, *L*_ds,max_, *L*(mature) and *L*(precursor) in the top three panels of Fig. 4. The performance of miRDP is significantly lower than that of miRA and miR-PREFeR, confirming results from [28].

It is interesting to note that in comparison to results for Chlamydomonas, the Arabidopsis precursor population is more homogeneous, showing a narrower length distribution and fewer complex secondary structures. The length distribution of mature miRNAs in Arabidopsis shows two characteristic peaks at 21 nt and 24 nt. Only a small fraction (∼15 %) of the loci corresponding to the 24 nt long sequences are repeat element-associated, which may support the association of these sequences with siRNAs/ta-siRNAs. This suggests that the majority of identified mature miRNAs of different lengths should be attributed to alternative miRNA biogensis pathways such as miRNA precursor processing by different members of the Dicer-like enzymes (see e.g. [16, 38]). The corresponding Volvocine distributions show the existence of a single peak at 21 nt. This suggests a change in or the absence of complex Dicer-like processing in green algae.

### Volvox carteri

We use miRA to identify novel miRNAs in the Volvocine organism *Volvox carteri* (Volvox). We use small RNA sequencing data derived from Volvox somatic cells during their vegetative cycle (GEO accession number GSE58703). Reads were mapped to the Volvox reference genome version 9.0 ([39]) using tophat/bowtie2. Distributions of key parameters equivalent to those discussed in the previous sections for Chlamydomonas and Arabidopsis are shown in the bottom panel of Fig. 4. A complete list of identified novel Volvox miRNAs is given in Table S4 of the Additional file 1.

To validate the identication of novel miRNAs in Volvox, Northern blots were performed on three randomly picked miRNAs from the list of identified novel miRNAs. Expression was confirmed for all three miRNA candidates, and the resulting blots are shown in Figure S3 of the Additional file 1.

The identified Volvox miRNAs show large similarities in the distribution of per-nucleotide free energy, and precursor and mature miRNA lengths compared to Chlamydomonas results. The slightly bi-modal distribution of MFE/nt suggests the existence of a plant-like and an algae-like miRNA sub-population, further confirming the existence of a heterogeneous population of miRNAs as was already the case for Chlamydomonas. Identified mature miRNA sequences show no similarity to identified mature miRNA sequences in Chlamydomonas. Given the large degree of similarity between the two genomes, the absence of any miRNA-conservation between the two closely related organisms is surprising, see also [21].

## Conclusion

miRA presents a new conservation-independent miRNA identification algorithm, which identifies genomic locations of miRNA precursors based on (small RNA) sequencing data of plants and plant-like organisms (algae). miRA is particularly suited to investigate heterogeneous miRNA precursor populations. Identification of miRNA precursors occurs through an evaluation of corresponding secondary structures and subsequent precursor processing accuracy. Our method has three key features: First, it allows for the identification of miRNAs in species with little or no miRNA conservation. Second, it enables a consistent investigation of both species-specific and homologous miRNAs in different organisms. Third, it allows for the identification of miRNA precursors with complex and heterogeneous secondary structures, such as precursors including e.g. additional sub-hairpins or multiple mature/star miRNA duplexes.

## Availability and requirements

**Project name:** miRA**Project home page:** https://github.com/mhuttner/miRA **Operating system(s):** Any Unix-based system (MacOS, Linux)**Programming language:** C**Other requirements:** Optional requirements: Java 1.6+, LaTeX, gnuplot**License:** GNU GPL

## Endnotes

^1^ RNAfold is invoked from the main program, with parameters being passed directly to RNAfold as part of miRA’s main routine.

^2^ Values given in the following sections correspond to default values, and may be changed by the user.

## Additional file

Additional file 1
**miRA default parameters.** (PDF 2333 kb)
