# Supplementary material for: miRA: adaptable novel miRNA identification in plants using small RNA sequencing data
Source: BMC Bioinformatics. 2015 Nov 5;16:370. doi: 10.1186/s12859-015-0798-3 (PMC4635600; doi:10.1186/s12859-015-0798-3)
Supplement: Additional file 1 — miRA default parameters. (PDF 2333 kb) [file 12859_2015_798_MOESM1_ESM.pdf]

miRA default parameters

Table S1: List of miRA default configuration parameters for different organisms (green algae and plants). Details about the various parameters are given in section **Method** of the main paper. See the miRA help (`miRA --help`) for details on how to call miRA for different organisms.

	Green algae	Plants
`openmp_thread_count`	10	10
`cluster_gap_size`	10	10
`cluster_min_reads`	10	10
`cluster_flank_size`	200	200
`cluster_max_length`	2000	2000
`min_precursor_length`	0	0
`max_precursor_length`	0	0
`max_mfe_per_nt`	-0.30	-0.20
`max_hairpin_count`	4	4
`min_double_strand_length`	18	18
`permutation_count`	100	100
`max_pvalue`	0.01	0.01
`min_coverage`	0.01	0.01
`min_paired_fraction`	0.55	0.55
`min_duplex_length`	18	18
`max_duplex_length`	30	30
`allow_three_mismatches`	1	1
`allow_two_terminal_mismatches`	0	0

Statistical significance testing

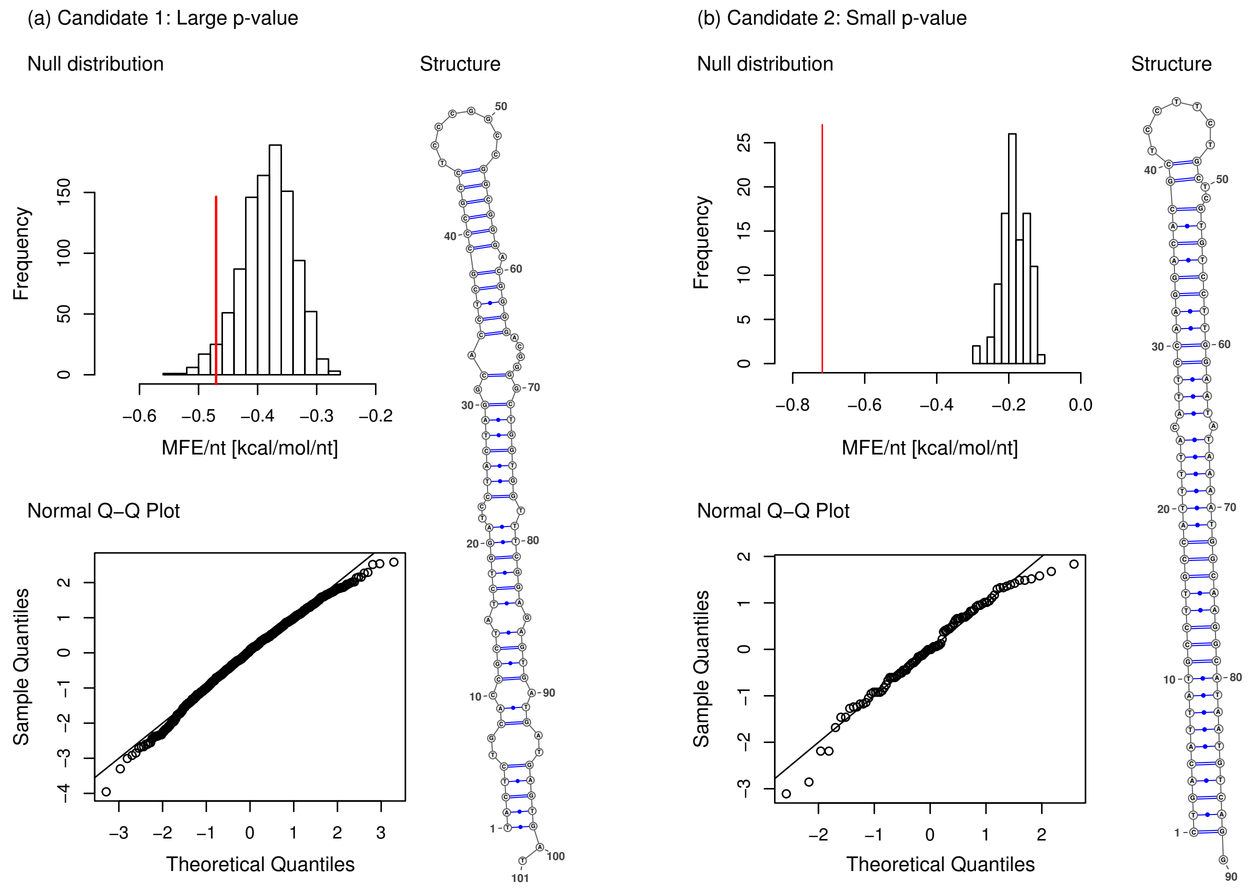

Figure S1: Two candidate miRNA precursors which pass the species-characteristic secondary structure constraints. Candidates that fail and pass the statistical significance test are shown in the left (a) and right (b) panels, respectively. For each candidate, (i) the per-nucleotide minimum free energy (MFE/nt) null distribution derived from random sequences with the same mono-nucleotide distribution including the candidate structure MFE/nt (vertical red line), (ii) the QQ plot for a normal distribution of MFE/nt, and (iii) the candidate structure are shown.

Flux simulator input parameters

Table S2: Flux Simulator input file used for generating *Chlamydomonas reinhardtii* mRNA background expression. Resulting reads were added to the simulated expression of 20 known miRNAs. For details see section **Results and discussion** from the main paper.

REF_FILE_NAME	cre_transcripts.gtf
GEN_DIR	scaffolds/
READ_LENGTH	35
TSS_MEAN	NaN
READ_NUMBER	15000000
NB_MOLECULES	5000000
GC_SD	0.1
GC_MEAN	0.5
SIZE_SAMPLING	AC
FRAG_SUBSTRATE	RNA
FRAG_METHOD	UR
FRAG_EZ_MOTIF	NlaIII
PAIRED_END	false
FASTA	true
TMP_DIR	tmp_folder/

Full list of identified miRNAs in *Chlamydomonas reinhardtii*

Table S3: Full list of 281 identified miRNAs in *Chlamydomonas reinhardtii* from miRA. Corresponding matches from Molnar et al. [1] are indicated. For details, see section **Results and discussion** in the main paper.

Name	Scaffold	Start	Stop	Match in Molnar et al.	Strand
cre-mir-1	scaffold_1	186331	186654	cr.01795s	plus
cre-mir-2	scaffold_1	757123	757188	–	–
cre-mir-3	scaffold_1	1273031	1273132	–	–
cre-mir-4	scaffold_1	1804913	1805129	cr.01801s	plus
cre-mir-5	scaffold_1	2346710	2346948	–	–
cre-mir-6	scaffold_1	2363527	2363598	–	–
cre-mir-7	scaffold_1	3204620	3204757	cr.01807s	plus
cre-mir-8	scaffold_1	3248133	3248410	–	–
cre-mir-9	scaffold_1	3943005	3943304	–	–
cre-mir-10	scaffold_1	3956391	3956682	–	–
cre-mir-11	scaffold_1	5142984	5143102	–	–
cre-mir-12	scaffold_1	5256108	5256473	cr.01820s	plus
cre-mir-13	scaffold_1	5256706	5257034	cr.01821	plus
cre-mir-14	scaffold_1	5463623	5463805	–	–
cre-mir-15	scaffold_1	6178959	6179370	–	–
cre-mir-16	scaffold_1	6221162	6221672	–	–
cre-mir-17	scaffold_1	6352152	6352512	cr.01824s	plus
cre-mir-18	scaffold_10	488235	488448	–	–
cre-mir-19	scaffold_10	543905	544044	–	–
cre-mir-20	scaffold_10	1416464	1417013	cr.01843s	plus
cre-mir-21	scaffold_10	1471975	1472151	–	–
cre-mir-22	scaffold_10	1589337	1589514	–	–
cre-mir-23	scaffold_10	1711642	1712044	cr.01846	plus
cre-mir-24	scaffold_10	1934031	1934143	cr.01847	plus
cre-mir-25	scaffold_10	1935777	1935927	cr.01848	plus
cre-mir-26	scaffold_10	2110160	2110214	–	–
cre-mir-27	scaffold_10	2282656	2282782	cr.01854s	plus
cre-mir-28	scaffold_102	95325	95708	–	–
cre-mir-29	scaffold_105	107839	107986	–	–
cre-mir-30	scaffold_11	454794	455016	–	–
cre-mir-31	scaffold_11	559504	559684	–	–
cre-mir-32	scaffold_11	562070	562299	–	–
cre-mir-33	scaffold_11	794783	794856	–	–
cre-mir-34	scaffold_11	1452352	1452689	–	–
cre-mir-35	scaffold_11	1700088	1700192	–	–
cre-mir-36	scaffold_110	47995	48144	–	–
cre-mir-37	scaffold_117	75991	76413	–	–
cre-mir-38	scaffold_118	47312	47371	–	–
cre-mir-39	scaffold_12	1259418	1259651	–	–
cre-mir-40	scaffold_12	1442771	1442841	–	–
cre-mir-41	scaffold_12	1460015	1460216	–	–
cre-mir-42	scaffold_12	1593161	1593266	–	–
cre-mir-43	scaffold_12	1998775	1998907	cr.01969	plus

Table S3 – *Continued from previous page*

Name	Scaffold	Start	Stop	Match in Molnar et al.	Strand
cre-mir-44	scaffold_12	2089149	2089286	–	–
cre-mir-45	scaffold_13	205866	206003	–	–
cre-mir-46	scaffold_14	1133579	1133683	–	–
cre-mir-47	scaffold_14	1390988	1391181	–	–
cre-mir-48	scaffold_14	1502891	1503016	–	–
cre-mir-49	scaffold_149	19446	19524	–	–
cre-mir-50	scaffold_15	112666	113203	–	–
cre-mir-51	scaffold_15	409124	409361	–	–
cre-mir-52	scaffold_15	416748	417061	–	–
cre-mir-53	scaffold_15	1291875	1292013	cr.02052	plus
cre-mir-54	scaffold_15	1918566	1918689	cr.02057	minus
cre-mir-55	scaffold_15	2058290	2058660	cr.02058	plus
cre-mir-56	scaffold_16	173311	173519	–	–
cre-mir-57	scaffold_16	815806	816012	–	–
cre-mir-58	scaffold_16	820250	820665	–	–
cre-mir-59	scaffold_16	837532	837807	–	–
cre-mir-60	scaffold_16	992901	993132	–	–
cre-mir-61	scaffold_16	1855015	1855075	–	–
cre-mir-62	scaffold_16	1919700	1919969	cr.02074	plus
cre-mir-63	scaffold_16	1920407	1920562	–	–
cre-mir-64	scaffold_17	363273	363376	–	–
cre-mir-65	scaffold_17	700848	701344	–	–
cre-mir-66	scaffold_17	755524	755590	–	–
cre-mir-67	scaffold_17	966146	966290	cr.02088	plus
cre-mir-68	scaffold_17	1366243	1366365	–	–
cre-mir-69	scaffold_17	1394541	1394862	–	–
cre-mir-70	scaffold_17	1522001	1522062	–	–
cre-mir-71	scaffold_17	1888384	1888558	–	–
cre-mir-72	scaffold_17	1980044	1980097	–	–
cre-mir-73	scaffold_17	2045477	2045616	–	–
cre-mir-74	scaffold_18	251264	251407	cr.02116	plus
cre-mir-75	scaffold_18	360474	360590	–	–
cre-mir-76	scaffold_18	697237	697344	–	–
cre-mir-77	scaffold_18	1349744	1349827	–	–
cre-mir-78	scaffold_18	1845607	1845672	–	–
cre-mir-79	scaffold_18	1879867	1880149	–	–
cre-mir-80	scaffold_19	455194	455331	–	–
cre-mir-81	scaffold_19	459639	459854	cr.02139s	plus
cre-mir-82	scaffold_2	982408	982634	–	–
cre-mir-83	scaffold_2	1742481	1742590	–	–
cre-mir-84	scaffold_2	2089105	2089504	–	–
cre-mir-85	scaffold_2	2333354	2333453	–	–
cre-mir-86	scaffold_2	3550938	3551241	–	–
cre-mir-87	scaffold_20	46389	46762	–	–
cre-mir-88	scaffold_20	352885	353199	–	–
cre-mir-89	scaffold_20	831488	831722	–	–
cre-mir-90	scaffold_20	917613	917757	–	–
cre-mir-91	scaffold_20	1015174	1015351	–	–

Table S3 – *Continued from previous page*

Name	Scaffold	Start	Stop	Match in Molnar et al.	Strand
cre-mir-92	scaffold_20	1892111	1892158	–	–
cre-mir-93	scaffold_21	332971	333087	–	–
cre-mir-94	scaffold_21	671512	671608	–	–
cre-mir-95	scaffold_21	673629	673862	–	–
cre-mir-96	scaffold_21	915988	916199	–	–
cre-mir-97	scaffold_21	1743159	1743380	–	–
cre-mir-98	scaffold_213	20096	20316	cr.02256	plus
cre-mir-99	scaffold_214	7189	7760	cr.02257	minus
cre-mir-100	scaffold_214	8982	9475	–	–
cre-mir-101	scaffold_214	10221	10462	–	–
cre-mir-102	scaffold_22	535228	535555	–	–
cre-mir-103	scaffold_22	1143939	1144217	–	–
cre-mir-104	scaffold_22	1315827	1315964	–	–
cre-mir-105	scaffold_23	107220	107452	–	–
cre-mir-106	scaffold_23	772588	772816	–	–
cre-mir-107	scaffold_23	884406	884532	–	–
cre-mir-108	scaffold_23	1270776	1271060	–	–
cre-mir-109	scaffold_24	753254	753395	–	–
cre-mir-110	scaffold_24	885605	885678	–	–
cre-mir-111	scaffold_24	1611658	1611774	–	–
cre-mir-112	scaffold_25	138596	138665	–	–
cre-mir-113	scaffold_25	325702	325799	–	–
cre-mir-114	scaffold_25	482296	482994	–	–
cre-mir-115	scaffold_25	996780	996956	–	–
cre-mir-116	scaffold_26	263656	263745	–	–
cre-mir-117	scaffold_26	422443	422543	–	–
cre-mir-118	scaffold_27	309617	309830	–	–
cre-mir-119	scaffold_27	990904	991156	–	–
cre-mir-120	scaffold_28	299413	299481	–	–
cre-mir-121	scaffold_28	688078	688278	–	–
cre-mir-122	scaffold_28	775440	775782	cr.02358s	plus
cre-mir-123	scaffold_28	1007553	1007626	–	–
cre-mir-124	scaffold_28	1039943	1040321	cr.02359s	plus
cre-mir-125	scaffold_28	1143676	1143832	–	–
cre-mir-126	scaffold_28	1424196	1424463	–	–
cre-mir-127	scaffold_283	8607	8746	–	–
cre-mir-128	scaffold_29	1174253	1174592	–	–
cre-mir-129	scaffold_3	93567	93760	–	–
cre-mir-130	scaffold_3	167739	167954	cr.02386	minus
cre-mir-131	scaffold_3	498410	498759	–	–
cre-mir-132	scaffold_3	1053384	1053864	–	–
cre-mir-133	scaffold_3	1150235	1150305	–	–
cre-mir-134	scaffold_3	1445075	1445122	–	–
cre-mir-135	scaffold_3	1535256	1535545	–	–
cre-mir-136	scaffold_3	1992199	1992410	–	–
cre-mir-137	scaffold_3	3062401	3062624	–	–
cre-mir-138	scaffold_30	1011524	1011689	–	–
cre-mir-139	scaffold_31	329920	330518	–	–

Table S3 – *Continued from previous page*

Name	Scaffold	Start	Stop	Match in Molnar et al.	Strand
cre-mir-140	scaffold_31	332427	333025	–	–
cre-mir-141	scaffold_31	368851	369227	–	–
cre-mir-142	scaffold_31	661225	661795	–	–
cre-mir-143	scaffold_31	1053150	1053211	–	–
cre-mir-144	scaffold_310	6806	6879	–	–
cre-mir-145	scaffold_32	259596	259776	cr.02446	plus
cre-mir-146	scaffold_32	655437	655645	–	–
cre-mir-147	scaffold_33	258259	258700	–	–
cre-mir-148	scaffold_330	492	543	–	–
cre-mir-149	scaffold_34	572892	572953	–	–
cre-mir-150	scaffold_35	109405	110018	–	–
cre-mir-151	scaffold_35	818350	818635	–	–
cre-mir-152	scaffold_35	929772	930108	cr.02466	minus
cre-mir-153	scaffold_35	1049290	1049550	–	–
cre-mir-154	scaffold_36	260699	260805	cr.02472s	plus
cre-mir-155	scaffold_36	503022	503133	–	–
cre-mir-156	scaffold_36	578006	578190	cr.02473	minus
cre-mir-157	scaffold_36	724530	724698	–	–
cre-mir-158	scaffold_37	408573	408860	–	–
cre-mir-159	scaffold_37	590564	591095	–	–
cre-mir-160	scaffold_37	784910	784984	–	–
cre-mir-161	scaffold_38	359786	359971	–	–
cre-mir-162	scaffold_38	360380	360531	–	–
cre-mir-163	scaffold_38	790373	790538	–	–
cre-mir-164	scaffold_38	857644	857925	–	–
cre-mir-165	scaffold_38	926469	926660	–	–
cre-mir-166	scaffold_39	459417	459469	–	–
cre-mir-167	scaffold_39	517788	517881	–	–
cre-mir-168	scaffold_39	656178	656390	–	–
cre-mir-169	scaffold_39	965155	965214	–	–
cre-mir-170	scaffold_4	752685	753098	–	–
cre-mir-171	scaffold_4	983312	983390	–	–
cre-mir-172	scaffold_4	1255613	1255912	–	–
cre-mir-173	scaffold_4	1655055	1655110	–	–
cre-mir-174	scaffold_4	1802578	1802654	–	–
cre-mir-175	scaffold_4	2037823	2038076	cr.02513s	plus
cre-mir-176	scaffold_4	2546564	2546731	–	–
cre-mir-177	scaffold_4	2555125	2555171	–	–
cre-mir-178	scaffold_4	2849916	2850081	–	–
cre-mir-179	scaffold_4	2941648	2941864	–	–
cre-mir-180	scaffold_4	2978267	2978438	–	–
cre-mir-181	scaffold_40	432517	432657	cr.02546	minus
cre-mir-182	scaffold_40	888731	889054	–	–
cre-mir-183	scaffold_41	199129	199234	–	–
cre-mir-184	scaffold_41	731110	731167	–	–
cre-mir-185	scaffold_41	760648	760974	–	–
cre-mir-186	scaffold_42	336177	336688	–	–
cre-mir-187	scaffold_42	638045	638103	–	–

Table S3 – *Continued from previous page*

Name	Scaffold	Start	Stop	Match in Molnar et al.	Strand
cre-mir-188	scaffold_44	373373	373744	–	–
cre-mir-189	scaffold_45	135429	135843	–	–
cre-mir-190	scaffold_45	178605	178988	–	–
cre-mir-191	scaffold_45	746353	746421	–	–
cre-mir-192	scaffold_46	42505	42655	–	–
cre-mir-193	scaffold_46	613342	613453	cr.02586	plus
cre-mir-194	scaffold_47	152162	152389	–	–
cre-mir-195	scaffold_48	281475	281919	–	–
cre-mir-196	scaffold_48	283336	283677	–	–
cre-mir-197	scaffold_49	113991	114355	–	–
cre-mir-198	scaffold_5	4526	4829	cr.02603	plus
cre-mir-199	scaffold_5	218028	218214	–	–
cre-mir-200	scaffold_5	339928	340221	–	–
cre-mir-201	scaffold_5	398845	399066	–	–
cre-mir-202	scaffold_5	455793	456209	–	–
cre-mir-203	scaffold_5	951388	951566	–	–
cre-mir-204	scaffold_5	1129922	1130321	–	–
cre-mir-205	scaffold_5	1507942	1508008	–	–
cre-mir-206	scaffold_5	2618828	2618992	–	–
cre-mir-207	scaffold_5	2621429	2621749	–	–
cre-mir-208	scaffold_50	21808	21978	–	–
cre-mir-209	scaffold_51	171276	171451	–	–
cre-mir-210	scaffold_51	235125	235474	–	–
cre-mir-211	scaffold_51	445199	445472	–	–
cre-mir-212	scaffold_51	446731	447170	–	–
cre-mir-213	scaffold_54	61882	62156	–	–
cre-mir-214	scaffold_54	88757	89154	–	–
cre-mir-215	scaffold_54	327842	327897	–	–
cre-mir-216	scaffold_54	459657	459746	–	–
cre-mir-217	scaffold_57	85276	85699	–	–
cre-mir-218	scaffold_58	46062	46224	–	–
cre-mir-219	scaffold_58	151233	151457	–	–
cre-mir-220	scaffold_58	451346	451615	–	–
cre-mir-221	scaffold_6	18946	19110	–	–
cre-mir-222	scaffold_6	90215	90327	–	–
cre-mir-223	scaffold_6	118493	118744	cr.02714	minus
cre-mir-224	scaffold_6	231365	231442	–	–
cre-mir-225	scaffold_6	1014100	1014405	cr.02719	minus
cre-mir-226	scaffold_6	1045064	1045234	–	–
cre-mir-227	scaffold_6	1649748	1649850	–	–
cre-mir-228	scaffold_6	1906386	1906522	–	–
cre-mir-229	scaffold_6	1960286	1960773	–	–
cre-mir-230	scaffold_6	1969723	1970208	cr.02725	plus
cre-mir-231	scaffold_6	2002313	2002494	–	–
cre-mir-232	scaffold_60	59802	60001	–	–
cre-mir-233	scaffold_60	281895	282025	–	–
cre-mir-234	scaffold_60	389236	389326	–	–
cre-mir-235	scaffold_61	269819	269902	–	–

Table S3 – *Continued from previous page*

Name	Scaffold	Start	Stop	Match in Molnar et al.	Strand
cre-mir-236	scaffold_61	326060	326121	–	–
cre-mir-237	scaffold_61	440245	440306	–	–
cre-mir-238	scaffold_64	413149	413312	–	–
cre-mir-239	scaffold_65	146187	146301	–	–
cre-mir-240	scaffold_656	7874	8068	–	–
cre-mir-241	scaffold_66	49053	49145	–	–
cre-mir-242	scaffold_7	151785	151895	–	–
cre-mir-243	scaffold_7	773094	773179	cr.02798s	plus
cre-mir-244	scaffold_7	1602863	1603103	–	–
cre-mir-245	scaffold_7	1694980	1695239	–	–
cre-mir-246	scaffold_7	2198524	2198638	–	–
cre-mir-247	scaffold_7	2270791	2270841	–	–
cre-mir-248	scaffold_70a	57279	57487	–	–
cre-mir-249	scaffold_70a	208269	208445	–	–
cre-mir-250	scaffold_71	96196	96385	–	–
cre-mir-251	scaffold_71	111419	111539	–	–
cre-mir-252	scaffold_73	111351	111594	–	–
cre-mir-253	scaffold_73	286771	286851	–	–
cre-mir-254	scaffold_74	277642	277897	–	–
cre-mir-255	scaffold_75	225319	225637	–	–
cre-mir-256	scaffold_75	274385	274871	–	–
cre-mir-257	scaffold_75	284493	284781	cr.02862s	plus
cre-mir-258	scaffold_75	368389	368849	–	–
cre-mir-259	scaffold_75	370855	371138	cr.02865	plus
cre-mir-260	scaffold_76	73490	73687	–	–
cre-mir-261	scaffold_76	233226	233364	cr.02870	plus
cre-mir-262	scaffold_78	77581	77727	–	–
cre-mir-263	scaffold_79	18267	18321	–	–
cre-mir-264	scaffold_79	84940	85003	–	–
cre-mir-265	scaffold_8	207172	207243	–	–
cre-mir-266	scaffold_8	1493157	1493214	–	–
cre-mir-267	scaffold_8	1677898	1678064	–	–
cre-mir-268	scaffold_8	1966126	1966230	–	–
cre-mir-269	scaffold_8	1966760	1966864	–	–
cre-mir-270	scaffold_8	2058567	2058738	–	–
cre-mir-271	scaffold_80	14465	15082	–	–
cre-mir-272	scaffold_84	96029	96378	–	–
cre-mir-273	scaffold_891	3252	3336	–	–
cre-mir-274	scaffold_9	7432	7567	–	–
cre-mir-275	scaffold_9	1364309	1364411	–	–
cre-mir-276	scaffold_9	2043913	2043958	–	–
cre-mir-277	scaffold_90	23732	23886	–	–
cre-mir-278	scaffold_94	194272	194410	–	–
cre-mir-279	scaffold_95	98807	98950	–	–
cre-mir-280	scaffold_95	143231	143461	–	–
cre-mir-281	scaffold_95	156838	157003	–	–

Examples of complex miRNA precursors in *Chlamydomonas reinhardtii*

Figure S2: Examples of complex miRNA precursors in *Chlamydomonas reinhardtii.* For details, see section **Results and discussion** in the main paper. Precursors often show expression profiles consistent with the production of multiple mature miRNA from the same precursor (example 1), as well as secondary structures containing additional hairpin loops (example 2). Coverage plots show the per-nucleotide read coverage for the plus (in red) and minus strand (in blue). Genomic positions and positions along the precursor sequence are given separately along the bottom and top x-axes. In the secondary structure plots, the primary (most strongly expressed) mature miRNA sequence is highlighted in red, and its complementary star sequence in blue.

Example 1: mir-cre-9

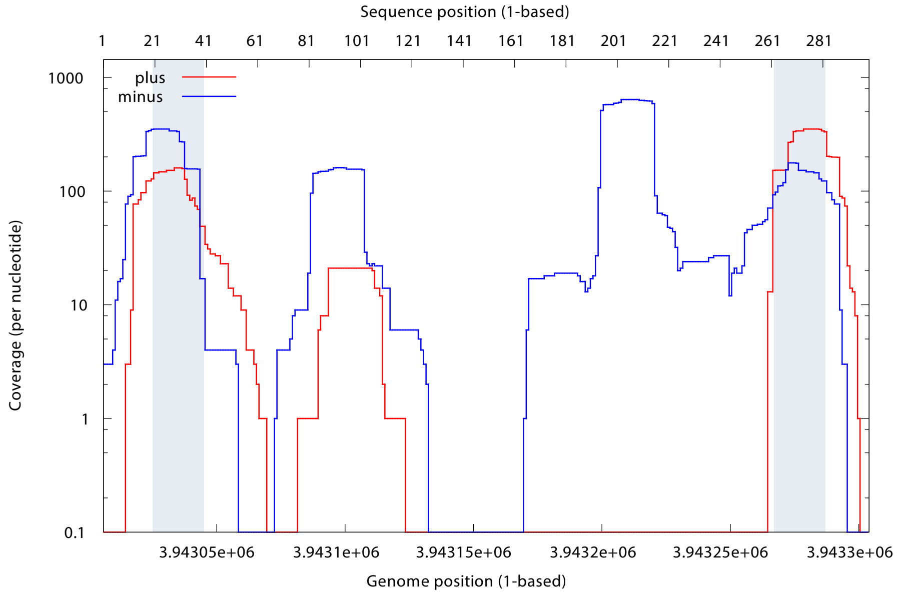

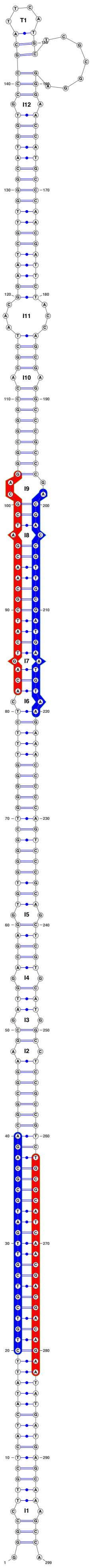

Example 2: cre-mir-14

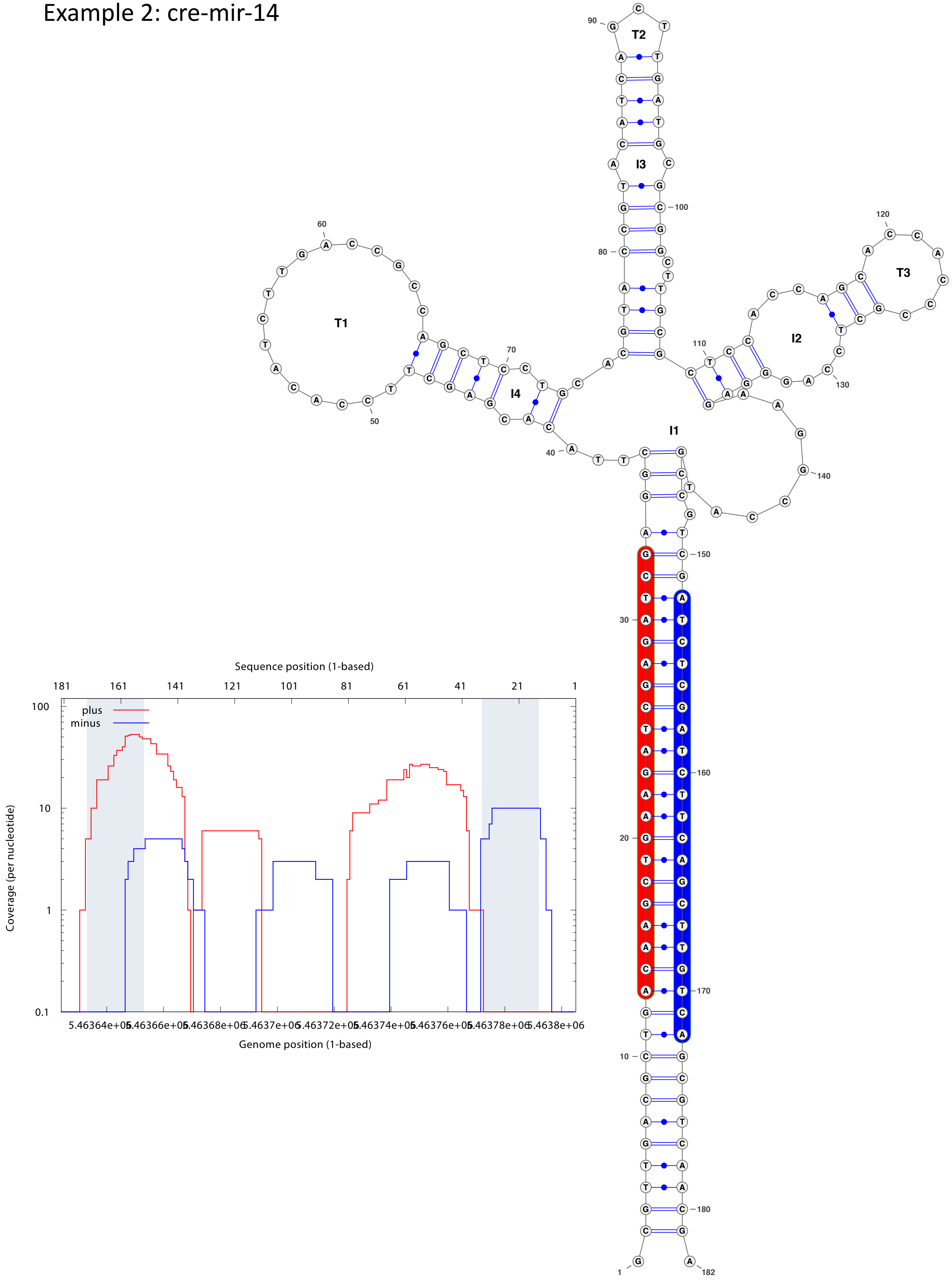

Full list of identified miRNAs in *Volvox carteri*

Table S4: Full list of 213 identified miRNAs in *Volvox carteri* from miRA. For details, see section **Results and discussion** in the main paper.

Name	Scaffold	Start	Stop	Strand
vca-mir-1	scaffold_1	3025666	3025841	plus
vca-mir-2	scaffold_1	3115004	3115278	minus
vca-mir-3	scaffold_1	3120004	3120203	plus
vca-mir-4	scaffold_1	3120004	3120203	minus
vca-mir-5	scaffold_1	3763375	3763592	minus
vca-mir-6	scaffold_1	4501926	4502219	plus
vca-mir-7	scaffold_1	4503209	4503443	plus
vca-mir-8	scaffold_1	4513619	4513956	plus
vca-mir-9	scaffold_1	4513637	4513960	minus
vca-mir-10	scaffold_1	7263654	7263960	plus
vca-mir-11	scaffold_1	7263655	7263961	minus
vca-mir-12	scaffold_1	10097708	10097959	plus
vca-mir-13	scaffold_1	11949608	11949820	plus
vca-mir-14	scaffold_1	14147874	14148043	plus
vca-mir-15	scaffold_100	67969	68168	minus
vca-mir-16	scaffold_100	67969	68187	plus
vca-mir-17	scaffold_101	5918	6017	minus
vca-mir-18	scaffold_101	5931	6004	plus
vca-mir-19	scaffold_101	6141	6379	plus
vca-mir-20	scaffold_11	1036056	1036256	plus
vca-mir-21	scaffold_11	2143080	2143398	minus
vca-mir-22	scaffold_11	2192683	2192771	plus
vca-mir-23	scaffold_11	2509131	2509452	plus
vca-mir-24	scaffold_11	2513057	2513256	minus
vca-mir-25	scaffold_11	2513057	2513257	plus
vca-mir-26	scaffold_11	2924104	2924325	minus
vca-mir-27	scaffold_11	2924121	2924342	plus
vca-mir-28	scaffold_115	11620	11803	minus
vca-mir-29	scaffold_115	11620	11803	plus
vca-mir-30	scaffold_115	19808	20019	plus
vca-mir-31	scaffold_118	13205	13416	plus
vca-mir-32	scaffold_12	484249	484353	plus
vca-mir-33	scaffold_12	484249	484354	minus
vca-mir-34	scaffold_12	516367	516757	plus
vca-mir-35	scaffold_12	775620	775836	plus
vca-mir-36	scaffold_13	238387	238650	minus
vca-mir-37	scaffold_13	1572532	1572645	minus
vca-mir-38	scaffold_13	1815683	1815876	minus
vca-mir-39	scaffold_13	1815703	1815856	plus
vca-mir-40	scaffold_14	1250672	1250763	plus
vca-mir-41	scaffold_14	1765799	1765992	plus
vca-mir-42	scaffold_14	1765802	1765989	minus
vca-mir-43	scaffold_14	1987966	1988211	minus
vca-mir-44	scaffold_14	1996254	1996453	plus

Table S4 – *Continued from previous page*

Name	Scaffold	Start	Stop	Strand
vca-mir-45	scaffold_14	2667584	2667787	minus
vca-mir-46	scaffold_14	2677641	2677775	plus
vca-mir-47	scaffold_14	2823445	2823644	minus
vca-mir-48	scaffold_14	2823445	2823644	plus
vca-mir-49	scaffold_15	123468	123554	minus
vca-mir-50	scaffold_15	1962855	1963181	minus
vca-mir-51	scaffold_15	1962928	1963117	plus
vca-mir-52	scaffold_16	1946027	1946250	minus
vca-mir-53	scaffold_16	2354023	2354122	plus
vca-mir-54	scaffold_17	1224	1483	minus
vca-mir-55	scaffold_17	1224	1483	plus
vca-mir-56	scaffold_17	589136	589570	minus
vca-mir-57	scaffold_17	589178	589527	plus
vca-mir-58	scaffold_17	2072270	2072371	plus
vca-mir-59	scaffold_17	2385974	2386092	minus
vca-mir-60	scaffold_172	10218	10429	plus
vca-mir-61	scaffold_18	12152	12416	minus
vca-mir-62	scaffold_18	12155	12412	plus
vca-mir-63	scaffold_18	1515867	1516000	plus
vca-mir-64	scaffold_18	1547269	1547500	minus
vca-mir-65	scaffold_18	2313251	2313453	minus
vca-mir-66	scaffold_18	2313251	2313471	plus
vca-mir-67	scaffold_19	242527	242822	plus
vca-mir-68	scaffold_19	337204	337554	plus
vca-mir-69	scaffold_19	607130	607397	minus
vca-mir-70	scaffold_19	607132	607399	plus
vca-mir-71	scaffold_19	625886	625982	minus
vca-mir-72	scaffold_19	1563079	1563322	plus
vca-mir-73	scaffold_192	13497	13708	minus
vca-mir-74	scaffold_2	5039346	5039726	minus
vca-mir-75	scaffold_20	541197	541303	minus
vca-mir-76	scaffold_20	559053	559155	minus
vca-mir-77	scaffold_20	862311	862419	minus
vca-mir-78	scaffold_20	1339809	1339892	plus
vca-mir-79	scaffold_20	2215647	2215846	minus
vca-mir-80	scaffold_21	113935	114140	minus
vca-mir-81	scaffold_21	133967	134092	plus
vca-mir-82	scaffold_21	181814	181945	plus
vca-mir-83	scaffold_21	428236	428288	minus
vca-mir-84	scaffold_21	2139205	2139550	minus
vca-mir-85	scaffold_21	2139209	2139572	plus
vca-mir-86	scaffold_22	2109078	2109183	minus
vca-mir-87	scaffold_23	117103	117303	minus
vca-mir-88	scaffold_23	990961	991166	minus
vca-mir-89	scaffold_23	1323711	1324019	minus
vca-mir-90	scaffold_23	1367272	1367576	plus
vca-mir-91	scaffold_25	951408	951499	plus
vca-mir-92	scaffold_25	1071163	1071405	plus

Table S4 – *Continued from previous page*

Name	Scaffold	Start	Stop	Strand
vca-mir-93	scaffold_25	1446715	1447018	minus
vca-mir-94	scaffold_25	1446718	1447015	plus
vca-mir-95	scaffold_26	1242759	1243037	plus
vca-mir-96	scaffold_26	1420743	1420905	minus
vca-mir-97	scaffold_26	1428370	1428615	plus
vca-mir-98	scaffold_27	824315	824405	plus
vca-mir-99	scaffold_27	1226683	1227081	plus
vca-mir-100	scaffold_27	1279043	1279375	plus
vca-mir-101	scaffold_27	1728267	1728558	plus
vca-mir-102	scaffold_28	169736	170150	minus
vca-mir-103	scaffold_3	77494	77609	minus
vca-mir-104	scaffold_3	82231	82339	minus
vca-mir-105	scaffold_3	87607	87708	plus
vca-mir-106	scaffold_3	97754	97862	minus
vca-mir-107	scaffold_3	139213	139321	minus
vca-mir-108	scaffold_3	155129	155237	minus
vca-mir-109	scaffold_3	554435	554786	plus
vca-mir-110	scaffold_3	554505	554891	minus
vca-mir-111	scaffold_3	1184724	1184842	plus
vca-mir-112	scaffold_3	1937439	1937543	plus
vca-mir-113	scaffold_3	4726162	4726380	minus
vca-mir-114	scaffold_31	9572	9826	minus
vca-mir-115	scaffold_31	452106	452305	minus
vca-mir-116	scaffold_31	452106	452324	plus
vca-mir-117	scaffold_31	691972	692175	minus
vca-mir-118	scaffold_31	691972	692192	plus
vca-mir-119	scaffold_31	713210	713523	plus
vca-mir-120	scaffold_31	1552230	1552328	plus
vca-mir-121	scaffold_32	39278	39376	minus
vca-mir-122	scaffold_32	832926	833027	plus
vca-mir-123	scaffold_32	1250240	1250345	minus
vca-mir-124	scaffold_321	2279	2530	plus
vca-mir-125	scaffold_321	6539	6736	minus
vca-mir-126	scaffold_321	6540	6736	plus
vca-mir-127	scaffold_33	884153	884439	plus
vca-mir-128	scaffold_38	65875	66093	plus
vca-mir-129	scaffold_38	275360	275547	plus
vca-mir-130	scaffold_38	288165	288270	minus
vca-mir-131	scaffold_38	359448	359649	plus
vca-mir-132	scaffold_38	594448	594794	plus
vca-mir-133	scaffold_39	716204	716362	minus
vca-mir-134	scaffold_4	224250	224506	minus
vca-mir-135	scaffold_4	2375489	2375563	plus
vca-mir-136	scaffold_4	3167788	3168030	minus
vca-mir-137	scaffold_4	3336855	3337037	minus
vca-mir-138	scaffold_4	3520094	3520455	plus
vca-mir-139	scaffold_4	3601379	3601597	plus
vca-mir-140	scaffold_4	4114603	4114936	plus

Table S4 – *Continued from previous page*

Name	Scaffold	Start	Stop	Strand
vca-mir-141	scaffold_41	458529	458781	plus
vca-mir-142	scaffold_42	98359	98556	plus
vca-mir-143	scaffold_45	745780	745868	plus
vca-mir-144	scaffold_47	10506	10717	minus
vca-mir-145	scaffold_47	84779	84979	minus
vca-mir-146	scaffold_47	236744	236906	minus
vca-mir-147	scaffold_47	237389	237493	minus
vca-mir-148	scaffold_47	256972	257191	minus
vca-mir-149	scaffold_47	256991	257190	plus
vca-mir-150	scaffold_48	145579	145696	minus
vca-mir-151	scaffold_49	596376	596476	minus
vca-mir-152	scaffold_5	788647	788882	minus
vca-mir-153	scaffold_5	3532896	3533138	minus
vca-mir-154	scaffold_5	3611611	3611927	minus
vca-mir-155	scaffold_5	3751466	3751719	minus
vca-mir-156	scaffold_5	4012909	4013178	minus
vca-mir-157	scaffold_51	123857	124099	plus
vca-mir-158	scaffold_51	123876	124081	minus
vca-mir-159	scaffold_51	577942	578066	plus
vca-mir-160	scaffold_52	157122	157285	minus
vca-mir-161	scaffold_54	535426	535531	minus
vca-mir-162	scaffold_56	431917	432264	plus
vca-mir-163	scaffold_58	276858	277139	plus
vca-mir-164	scaffold_58	276877	277122	minus
vca-mir-165	scaffold_58	367025	367187	minus
vca-mir-166	scaffold_58	367025	367187	plus
vca-mir-167	scaffold_6	842730	843033	plus
vca-mir-168	scaffold_6	931107	931365	minus
vca-mir-169	scaffold_6	1377954	1378242	minus
vca-mir-170	scaffold_61	371712	371958	minus
vca-mir-171	scaffold_61	372089	372166	minus
vca-mir-172	scaffold_63	278358	278625	plus
vca-mir-173	scaffold_63	278358	278625	minus
vca-mir-174	scaffold_7	12327	12538	minus
vca-mir-175	scaffold_7	242533	242635	minus
vca-mir-176	scaffold_70	142310	142521	plus
vca-mir-177	scaffold_71	22645	22743	minus
vca-mir-178	scaffold_72	37063	37283	plus
vca-mir-179	scaffold_72	54385	54628	minus
vca-mir-180	scaffold_74	244784	244870	plus
vca-mir-181	scaffold_74	256120	256353	plus
vca-mir-182	scaffold_76	165394	165519	plus
vca-mir-183	scaffold_8	379884	380141	minus
vca-mir-184	scaffold_8	1277759	1278126	minus
vca-mir-185	scaffold_8	1277835	1278161	plus
vca-mir-186	scaffold_8	1385271	1385622	plus
vca-mir-187	scaffold_8	1385676	1386002	plus
vca-mir-188	scaffold_8	2206685	2206890	minus

Table S4 – *Continued from previous page*

Name	Scaffold	Start	Stop	Strand
vca-mir-189	scaffold_8	2206707	2206889	plus
vca-mir-190	scaffold_81	102237	102517	plus
vca-mir-191	scaffold_84	21462	21652	minus
vca-mir-192	scaffold_84	73479	73584	minus
vca-mir-193	scaffold_84	117100	117320	minus
vca-mir-194	scaffold_84	117120	117319	plus
vca-mir-195	scaffold_85	25755	25859	minus
vca-mir-196	scaffold_85	115486	115764	minus
vca-mir-197	scaffold_85	115488	115764	plus
vca-mir-198	scaffold_85	195028	195327	minus
vca-mir-199	scaffold_85	197027	197207	plus
vca-mir-200	scaffold_86	124094	124227	plus
vca-mir-201	scaffold_86	124477	124649	minus
vca-mir-202	scaffold_9	3505583	3505767	plus
vca-mir-203	scaffold_90	11903	12102	minus
vca-mir-204	scaffold_93	137718	137930	plus
vca-mir-205	scaffold_93	140763	140890	plus
vca-mir-206	scaffold_936	1719	1930	plus
vca-mir-207	scaffold_94	1933	2102	plus
vca-mir-208	scaffold_94	42923	43013	plus
vca-mir-209	scaffold_94	146810	146915	minus
vca-mir-210	scaffold_95	21817	22000	plus
vca-mir-211	scaffold_95	28955	29154	minus
vca-mir-212	scaffold_95	75892	76111	minus
vca-mir-213	scaffold_97	123142	123240	plus

Validation of putative miRNAs in *Volvox carteri*

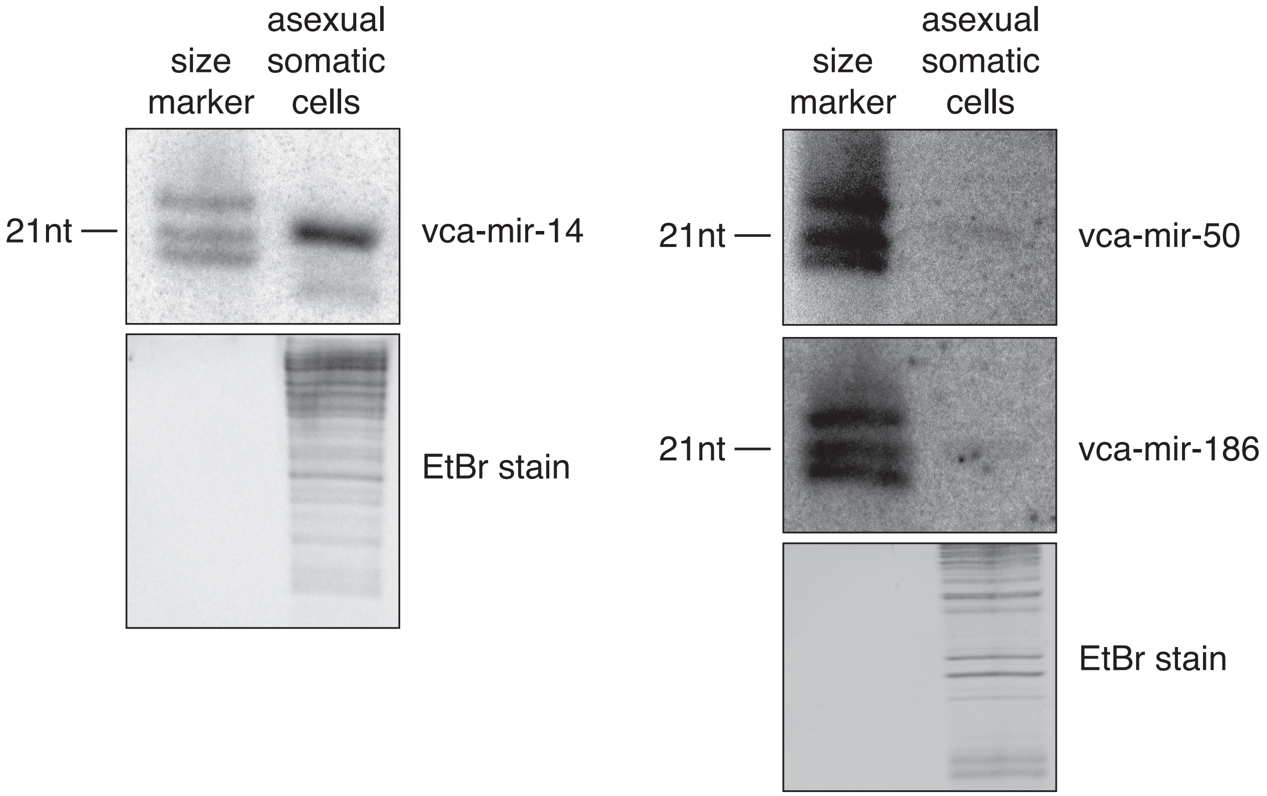

Figure S3: Total RNA from somatic cells was isolated and Northern blots were performed on randomly picked miRNA candidates from Table S4. A radioactively labeled size marker with the sizes of 19 nt, 21 nt and 24 nt was loaded as reference. Lower panel shows the ethidium bromide stain of the corresponding gel to control RNA integrity.

References

[1] Attila Molnár, Frank Schwach, David J Studholme, Eva C Thuenemann, and David C Baulcombe. miRNAs control gene expression in the single-cell alga Chlamydomonas reinhardtii. *Nature*, 447(7148):1126–1129, May 2007.
